# Drug advertisements in daily newspapers: a case study with the Hannoversche Allgemeine Zeitung (Hannover General Newspaper)

**DOI:** 10.1007/s00210-025-04015-z

**Published:** 2025-03-21

**Authors:** Ghaith Wafai, Roland Seifert

**Affiliations:** Institute of Pharmacology, Hannover Medical School, D-30625 Hannover, Germany

**Keywords:** Drug advertising, Therapeutic Products Advertising Act (Medicines advertising law), Hannoversche Allgemeine Zeitung, Pharmacy review

## Abstract

The Hannoversche Allgemeine Zeitung (HAZ, The Hannover General Newspaper) was launched in 1949 and is the largest daily newspaper in Lower Saxony, Germany. Drug advertisements in daily newspapers are common but have not yet been the topic of scientific investigation. This study analyzed all 181 medical advertisements published in the HAZ issued in 2021, all of which promoted over-the-counter medicines. Information was extracted from each advertisement and checked for selected parameters relating to the general structure of an advertisement, the content of the advertisement and the medical products themselves. The Therapeutic Products Advertising Act (Medicines Advertising Law; Heilmittelwerbegesetz (HWG)) of 1965 regulates the handling of medical advertisements through mandatory information and prohibitions. All advertisements were checked for compliance with the Act. The Therapeutic Products Advertising Act is loosely worded and poorly implemented, i.e., in only 52% of the advertisements mandatory information was included and only 69% of the advertisements avoided the prohibitions of the law. Furthermore, there is a clear connection between the newspaper’s readership, common diseases in the German population and the indications for the preparations. The most common indication was joint pain with 22.65%, this correlates with the prevalence of the disease in Germany and the age of the newspaper’s readership. In most cases, scientific evidence of the effectiveness of the preparations was lacking and only 46 of the 181 advertisements mention clinical studies. All in all, this paper gives an insight into the world of medical advertisements in a German newspaper, where marketing mechanisms, the kind of the consumer and the prices of the advertisement play a far more bigger role than scientific evidence. The Medicines Advertising Law has many flaws that are exploited by the drug companies to address the readers of the newspaper. The law needs to be stricter and better controlled to protect the layman from false medical information.

## Introduction

The Hannoversche Allgemeine Zeitung (HAZ) was founded by Erich Madsack on August 25, 1949. From its inception, HAZ benefited from the readership of its predecessor, the Hannoverscher Anzeiger, which was founded by August Madsack (1856–1933) and first published on February 28, 1893. Today, HAZ is the largest daily newspaper in Lower Saxony. It is published from Monday to Saturday, with a daily circulation of 126,718 copies, of which 19,247 (14.8%) are in digital format, and a readership of 332,000 (ma Tageszeitungen 2022) https://drive.google.com/file/d/1B7hWjm3PubLAmJOevhiVBOAbGztSv3FC/view?usp=sharing. Online subscriptions for HAZ have been increasing rapidly, with a remarkable growth of 364.19% observed between January 2020 and January 2022 (ma Tageszeitungen 2022). Drug advertisement is common in daily newspapers but rarely studied in scientific studies. Apart from a recent study on drug advertising in the German free-of-charge health magazine Apotheken Umschau (Pharmacy Review), no other studies could be found on drug advertising in German public newspapers. However, recent studies have dealt with medical advertising in other German media. A study reviewed the compliance of the HWG (Heilmittelwerbegesetz (Medicines Advertising law)) in advertising for products in online pharmacies (Barlage and Seifert 2024), another study examined advertisements for prescription-free drugs and dietary supplements in the professional journal Deutsche Apotheker Zeitung (German Pharmacy Journal) (Kuschel and Seifert 2024), and lastly, a study looked at medical advertisements in German television (Philipp and Seifert 2024). The studies consistently show the lack of proper implementation of the HWG and the targeting of the specific consumer through advertising methods.

Pharmaceutical companies are increasingly promoting their medical products and direct-to-consumer-advertising (DTCA) has become very common in the last decade especially with the big variety of marketing options (So and Kim 2024). There has been an obvious shift in the marketing strategy of drug manufacturers towards providing information about medical products directly to the consumer rather than healthcare professionals, thus DTCA spending has increased by 460% from 1997 to 2016 and DTCA is changing the patient-physician relationship from a professional model, which relies on a knowledgeable intermediary with fiduciary responsibility, to a consumer model, where patients approach physicians and pharmacists with preconceived ideas about the services they desire (Franquiz and McGuire 2021). The Apotheken Umschau (Pharmacy Review) study examined drug advertising in the magazine across all 48 issues published in 2020 and 2021. The study shows how psychological factors and marketing methods play a bigger role in the types of ads featured in the magazine, overshadowing the pharmacological aspects of the medical products. Specific scientific studies are cited in only 5% of the analyzed products (Keuper and Seifert 2024). The Heimittelwerbegesetz “HWG” (Therapeutic Products Advertising Act or the Act on Advertising in the Healthcare Sector) was introduced in 1965. The law regulates misleading advertising of medical products. The law distinguishes between advertising for professionals with prescription drugs and advertising for laypersons with over-the-counter drugs. The law has 18 paragraphs and an annex to § 12. Paragraphs § 4 and § 3 summarize the mandatory information and prohibitions in an advertisement. Failure to comply with this can be classified as a criminal offense. The HWG was last updated in 2012 (Fritsche 2021). The Hannoversche Allgemeine Zeitung “HAZ” has many readers every day in Lower Saxony, and the medical advertisements in the magazine could impact their health.

Our present study aims to gain a better understanding of how these advertising mechanisms work. Additionally, the implementation of the Medicines Advertising Law was reviewed, as HAZ’s internal testing mechanisms for medical ads are not accessible to outsiders.

## Material and methods

To provide a framework for this study, the year 2021 and the HAZ print newspapers were selected. One hundred eighty-one medical advertisements were analyzed. These included thirty-nine products for which advertisements could be found. These are all the medical advertisements found in the year 2021; none was excluded.

Access to all HAZ newspapers from 2021 was facilitated by the Hannover City Library, which archives HAZ issues and other newspapers from various years. By putting microfilms in a scanner, the newspapers from January, February, March, and April were browsed using specialized software. Screenshots of all the medical advertisements were saved and documented for later analysis. The Hannover City Library also pointed out that HAZ issues are accessible on the website [PressReader], allowing the remaining issues from other months to be viewed and medical advertisements documented. Understanding the readership structure of HAZ was also very important to potentially link it to the medical advertisements. According to (ma Tageszeitungen 2022) https://drive.google.com/file/d/1B7hWjm3PubLAmJOevhiVBOAbGztSv3FC/view?usp=sharing 69% of readers are over 40 years old, with a gender distribution of 52% men and 48% women. Additionally, 58% of readers have a net household income of more than € 3,000, 89% are employed or retired, and 83% have no children under the age of 14 in the household.

Afterwards, certain parameters were selected based on which the advertisements could be analyzed. For this purpose, Excel tables (raw data tables) were created to be able to enter the information extracted from each individual advertisement. Three main parameter groups emerged:

- General information of the advertisements: Indication area, day of publication, images in the advertisement, size of the advertisement, the presence of pharmacy leaflets, reference to website, page of the advertisement, position of the advertisement on the page and size of the advertisement. Content of the advertisement: question in the headline, explanation of the symptoms and causes of the disease, explanation of the active ingredients of the drug, type of drug, patient experience, mention of side effects and chronic risk cases, and reference to studies.
- Preparations: manufacturer, dosage form, defined daily doses (DDD), daily therapy costs, and trade name meaning.

**Fig. 1:**
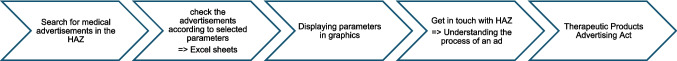
Analysis of drug advertising in the HAZ, shown in a flow chart

These data were then presented in graphs. In addition, the HAZ was contacted to develop an understanding of how the manufacturers can advertise their products (Fig. 1). Finally, a closer analysis of the implementation of the HWG was carried out. The analysis was carried out using two Excel tables with mandatory information and prohibitions of the law.

## Results

### Overview of the key features of all advertisements

Table 1 presents the key features of each product in all the medical advertisements. From the table, it becomes apparent that 8 companies were responsible for the 181 medical advertisements in the HAZ in 2021, promoting a total of 39 products. The product with the highest number of advertisements (16 ads) was product 14, which is indicated for joint pain. This was followed by product 7, indicated for nerve/back pain, and then product 12, which also indicated for joint pain, with 12 advertisements.

**Table 1:** Overview of the key features of all advertisements

	Indication	Number of ads	Daily therapy costs (acute)	Company	Pharmaceutical form of the product	Drug class
Product 1	Sexual weakness	6	2.47 €	Company 1	Liquid drops	Homeopathic drug
Product 2	Sexual weakness	6	9.90 €	Company 1	Tablet form	Homeopathic drug
Product 3	Sexual weakness	5	1.43 €	Company 1	Capsule form	Nutritional supplements
Product 4	Nerve/back pain	5	3.49 €	Company 1	Tablet form	Homeopathic drug
Product 5	Nerve/back pain	12	1.35 €	Company 4	Liquid drops	Homeopathic drug
Product 6	Nerve/back pain	2	0.87 €	Company 4	Capsule form	Nutritional supplements
Product 7	Nerve/back pain	14	1.19 €	Company 2	Liquid drops	Homeopathic drug
Product 8	Prostate complaints	4	1.25 €	Company 1	Liquid drops	Homeopathic drug
Product 9	Dizziness	6	2.37 €	Company 1	Liquid drops	Homeopathic drug
Product 10	Joint pain	11	2.25 €	Company 1	Liquid drops	Homeopathic drug
Product 11	Joint pain	6	1.45 €	Company 1	Powder form	Nutritional supplements
Product 12	Joint pain	5	1.24 €	Company 1	Liquid drops	Homeopathic drug
Product 13	Joint pain	2	Not calculable	Company 1	Gel	Homeopathic drug
Product 14	Joint pain	16	1.76 €	Company 2	Tablet form	Plant-based
Product 15	Constipation/flatulence	5	Not calculable	Company 3	Granule form	Nutritional supplements
Product 16	Irritable bowel and diarrhea	11	2.40 €	Company 3	Capsule form	Probiotics
Product 17	Irritable stomach	5	4.02 €	Company 3	Capsule form	Nutritional supplements
Product 18	Skin/facial disorder	3	2.14 €	Company 3	Powder form	Nutritional supplements
Product 19	Skin/facial disorder	3	1.89 €	Company 6	Drinkable collagen solution	Nutritional supplements
Product 20	Hair	1	Not calculable	Company 5	Shampoo and conditioner	Nutritional supplements
Product 21	Hair	2	1.50 €	Company 1	Liquid drops	Homeopathic drug
Product 22	Skin/facial disorder	3	Not calculable	Company 6	Cream	Cream
Product 23	Immune system boost	2	1.88 €	Company 3	Sachets	Probiotics
Product 24	Immune system boost	4	2.90 €	Company 1	Oil	Nutritional supplements
Product 25	Strained muscles	10	Not calculable	Company 1	Gel	Gel
Product 26	rheumatic pain	8	Not calculable	Company 2	Cream	Plant-based
Product 27	(Corona) Mouth and throat wash	3	Not calculable	Company 7	Spray	Antiviral
Product 28	Hemorrhoids	5	1.10 €	Company 1	Liquid drops	Homeopathic drug
Product 29	Weight loss	1	0.48 €	Company 2	Liquid drops	Homeopathic drug
Product 30	Diabetes	5	0.81 €	Company 2	Liquid drops	Homeopathic drug
Product 31	Memory and concentration problems	1	0.75 €	Company 1	Liquid drops	Homeopathic drug
Product 32	Water retention and leg pain	1	3.36 €	Company 2	Tablet form	Plant-based
Product 33	Painkillers	1	1.27 €	Company 1	Tablet form	Analgesics
Product 34	Painkillers	1	1.95 €	Company 1	Capsule form	Analgesics
Product 35	Lack of sleep	1	0.43 €	Company 1	Dragee	Plant-based
Product 36	Migraine	2	5.50 €	Company 1	Tablet form	Analgesics
Product 37	(Corona) Mouth and throat wash	1	1.20 €	Company 5	Spray	Antiviral
Product 38	Joint pain	1	Not calculable	Company 8	Gel	Analgesics
Product 39	Vaginal dryness	1	Not calculable	Company 7	Cream	Cream

### Indication areas of the advertisements

**Fig. 2:**
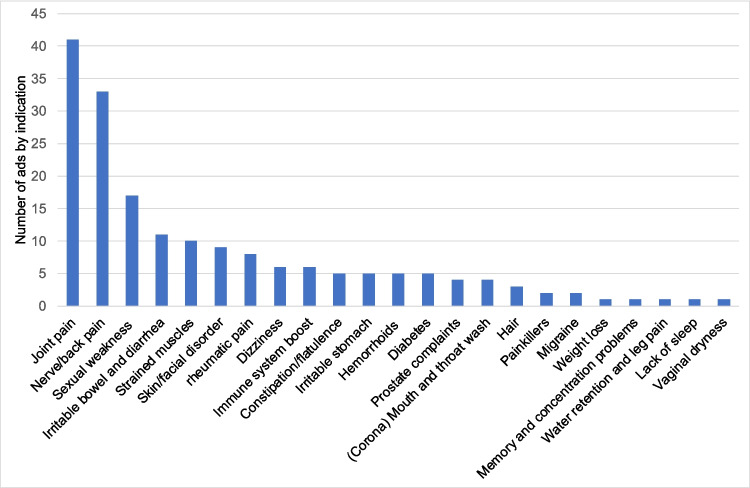
Analysis of all 181 advertisements by indication area. The data are shown in a bar chart

Figure 2 shows the number of indications occurring for each indication area. The most common indication was joint pain (41) followed by nerve/back pain (33) and sexual weakness (17) since sexual dysfunction affects both men and women, and the HAZ readership is nearly evenly split between men (52%) and women (48%) (ma Tageszeitungen 2022), the topic holds relevance for its entire audience. Population-representative surveys in Germany report a prevalence of one or more sexual problems of 33.4% in men and 45.7% in women (Brenk-Franz et al. 2023). The indication area weight loss was represented only once although 53.5% of adults in Germany are affected by overweight, and the prevalence of obesity is 19% (Schienkiewitz et al. 2022). Poor memory and concentration, water retention and abdominal pain, and lack of sleep and vaginal dryness were also only represented once.

### Number of ads by day of publication

**Fig. 3:**
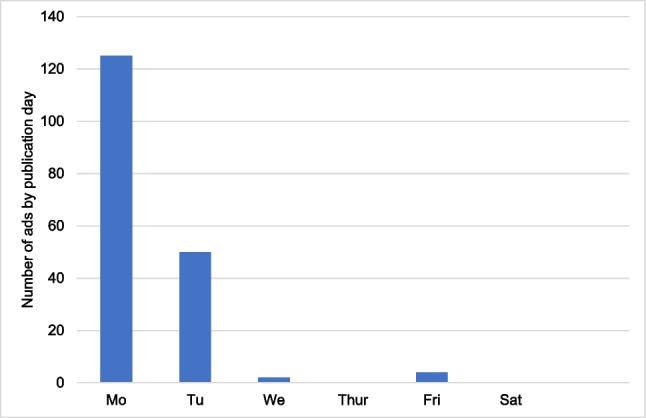
Representation of all analyzed ads in relation to their publication days. The data are presented in a bar chart

Figure 3 shows the total of 181 advertisements in relation to the day of the week on which the advertisement was published. Most ads were published on Monday (125) and Tuesday (50). In contrast, there were no ads on Thursdays and Saturdays. This can be attributed to the fact that, aside from the type of advertisement, placing an ad in the HAZ is less expensive during the week than on weekends. Additionally, these are medical advertisements that often serve to remind readers of their health concerns. On weekends, however, most people prefer to relax and enjoy their free time, and many stores that sell the advertised products are typically closed.

### Manufacturers of the preparations

**Fig. 4:**
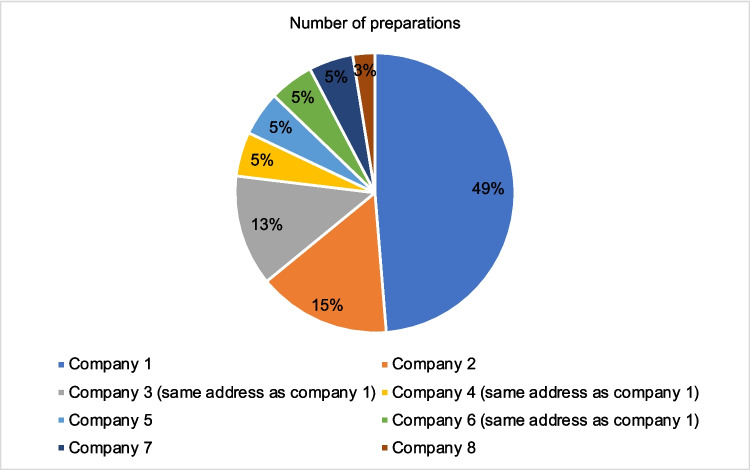
The pie chart shows the number of preparations by manufacturer as a percentage

Only 8 manufacturers were responsible for all 181 advertisements (39 products) in the HAZ in 2021 (Table 1). Of these, 72% of the products were produced by company no. 1 or companies 3, 4, 5, which have the same address as company no. 1 (Fig. 4). In contrast company 8 advertised for a single product.

**Fig. 5:**
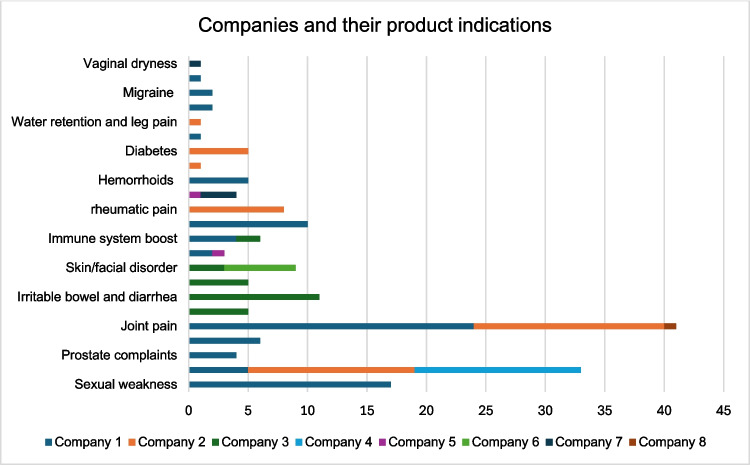
The bar chart illustrates the indication areas of the products for each company

Figure 5 illustrates the indication areas of the products for each company. Company no. 1 dominated the medical advertising landscape in 2021, covering a wide range of indication areas, particularly joint pain, sexual weakness, and strained muscles, as well as other areas such as hair loss, nerve and back pain, prostate issues, hemorrhoids, and more. Company no. 2, on the other hand, focused its advertising efforts primarily on nerve and back pain, joint pain, and rheumatic conditions. Company no. 3 focused its ads predominantly on indications related to the gastrointestinal tract. Company no. 4’s advertising was limited to nerve and back pain. Companies 5, 6, 7, and 8 had the fewest ads, with their indication areas represented by only one or two products addressing one or two specific indications.

### Drug class

**Fig. 6:**
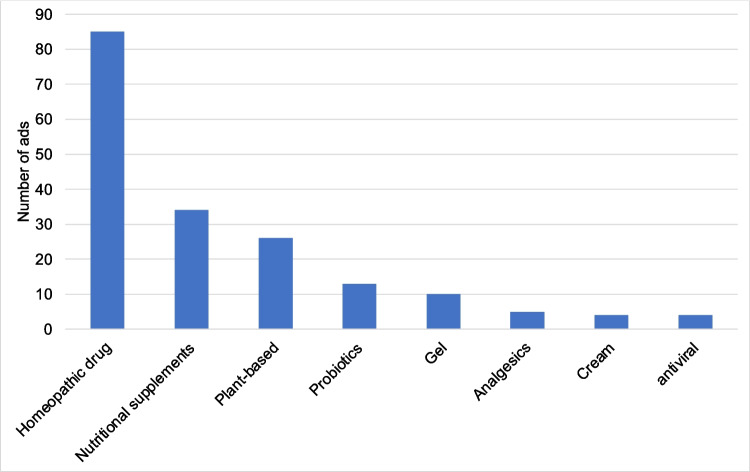
The bar chart shows the indications according to their drug class

According to § 4 of the HWG, the class of the medicinal product must be mentioned in every advertisement. Pursuant to §10 of the HWG “Prescription-only medicinal products may only be advertised to doctors, dentists, veterinarians, pharmacists and persons who trade in these medicinal products by law” (https://www.gesetze-im-internet.de/heilmwerbg/). Accordingly, none of the 181 advertisements contained prescription-only medicines. Most of the advertisements (85) were for homeopathic preparations as the DDD (defined daily dose) costs for homeopathic medicines haven been increasing over the years and are often more expensive than the rational pharmacological alternative (Leemhuis and Seifert 2024). Food supplements (34) and herbal preparations (26) were also commonly advertised (Fig. 6). Drugs with the fewest advertisements were analgesics, creams, and antiviral drugs (Fig. 6).

Among the 181 advertisements, 6 were for immune boosters and only 4 for coronavirus disease 2019 (COVID-19) mouth and throat rinses. This indicates that the pandemic of COVID-19 had not had a major impact on the advertisements in the HAZ in 2021. The likelihood of the manufacturers being able to advertise preparations related to COVID-19 was very low, in line with the speed of the spread of the pandemic, which is why we could hardly see any corona preparations in the advertisements.

### Daily therapy costs

The source of the product prices was the website (www.shop-apotheke.com). The acute and chronic defined daily doses (DDD) recommended by the manufacturer were crucial for calculating the daily therapy costs. Prices for gels and creams could not be calculated. The daily therapy costs were calculated for both acute and chronic therapy. For drop preparations, the price of a single drop was determined and multiplied by the recommended DDD. It was established that 1 mL of liquid equals 20 drops (2024, drops (liquid), Wikipedia).

**Fig. 7:**
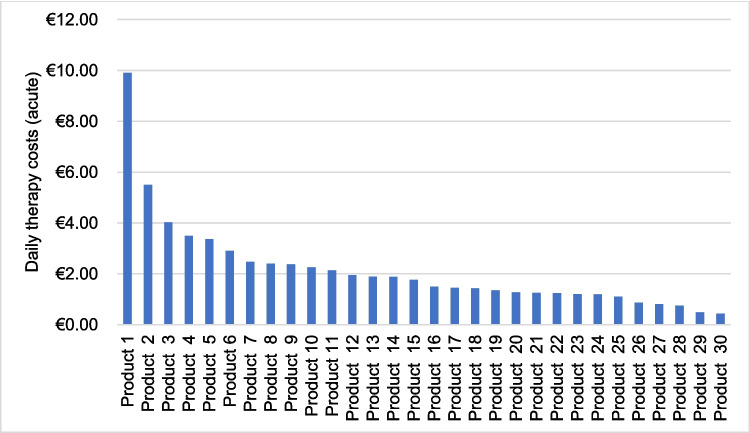
The bar chart shows the daily treatment costs of 30 preparations for an acute DDD

Prices for 30 out of 39 preparations included in the 181 advertisements could be calculated for acute therapy (Fig. 7). The mean value for acute therapy costs was € 2.15 with a standard deviation of 1.83. A preparation indicated for “sexual weakness” had the highest average acute daily treatment cost at € 9.90. The preparations with the lowest daily costs were for “lack of sleep,” “weight loss,” and “memory impairment.”

**Fig. 8:**
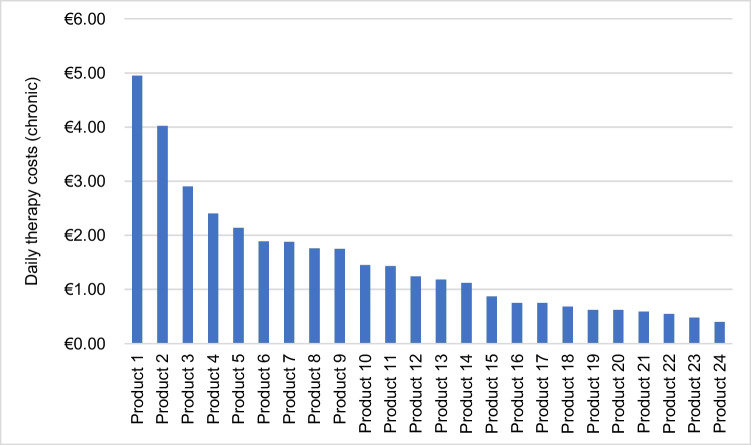
The bar chart shows the daily treatment costs of 24 preparations for a chronic DDD

For chronic therapy, the daily treatment costs could be calculated for only 24 preparations, as no recommended chronic DDD was specified by the manufacturer for the others (Fig. 8). The mean value for chronic daily treatment costs was €1.52 with a standard deviation of 1.14. The same drug had the highest average chronic daily treatment cost at € 4.95. The preparations with the lowest daily costs were for the indications “diabetes,” “weight loss,” and “hemorrhoids.”

### Proof of studies on the preparations

**Fig. 9:**
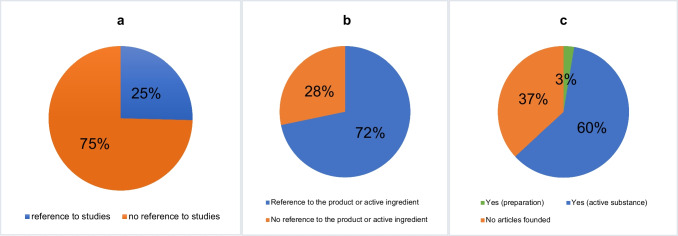
Review of the studies mentioned in the advertisements; **a** reference to studies in the ads; **b** relevance of the study for the preparation; **c** PubMed articles on preparations

A key focus of this study was to assess the extent to which the effectiveness of the 39 products mentioned in the 181 advertisements has been proven by scientific studies. Studies were mentioned in 46 advertisements (25%) (Fig. 9a). These studies were further analyzed to determine whether they referred to the specific product or to substances contained in the product. Thirty-three (72%) of the studies addressed the product itself or one of its active ingredients (Fig. 9b). We also investigated whether PubMed entries could be found for the 39 individual products or their active substances. A PubMed article was identified for only one product. For 23 preparations, articles were found on the active ingredients contained in the products. No articles were found for 14 products (Fig. 9c).

To evaluate the credibility of the 33 studies, they were divided into evidence classes as outlined by Mehrholz (2010). Class 1a includes the studies that underwent a systematic review based on methodologically high-quality controlled randomized trials (RCTs). Class 1b encompasses studies that include a sufficiently large, methodologically high-quality RCT. Class 2a comprises high-quality studies without randomization, such as cohort study. Class 2b contains high-quality studies of a different type of quasi-experimental study. Class 3 contains methodologically high-quality non-experimental studies. Finally, class 4 includes studies based on the opinions and convictions of respected authorities (from clinical experience), expert commissions, descriptive studies, and case studies.

**Fig. 10:**
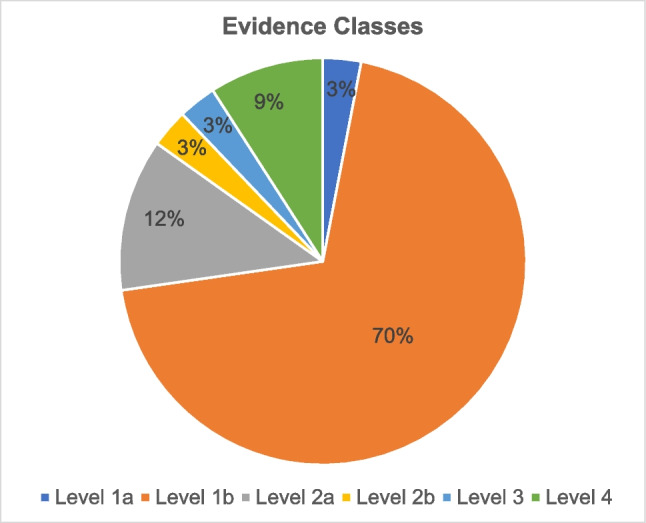
Evidence classes of mentioned studies in the 181 ads. The numbers refer to the 72% of “yes” studies with reference to preparation or active ingredient (a total of thirty-three studies) in Fig. 9b

Figure 10 presents the classification of the 33 studies by evidence classes. Seventy percent of the studies fell into class 1b. Only one study qualified for class 1a, while 3 studies were classified as class 4.

### Implementation of the Therapeutic Products Advertising Act

A traffic light system was developed to monitor the implementation of the HWG. The green color indicates compliance, yellow for controversial, and red for non-compliance. Two separate Excel tables were created to document information from the ads in relation to HWG requirements.

Table 2 addresses the mandatory information in a medical advertisement for outside specialist circles in accordance with Section 4 of the HWG (Fritsche 2021). This information includes the name of the company, registered office of the company, drug name, warnings, contraindications, area of application, the text “For risks and side effects, read the package leaflet and ask your doctor or pharmacist” (Zu Risiken und Nebenwirkungen lesen Sie die Packungsbeilage und fragen Sie Ihren Arzt oder Apotheker), and the composition of the drug.

**Table 2:**
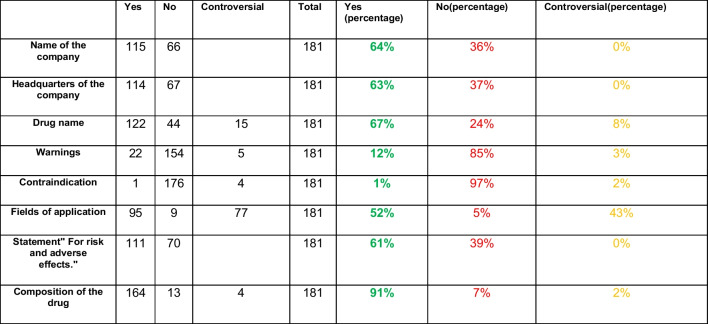
Implementation of the displays for the mandatory information according to the HWG. Absolute numbers and percentages are shown for each parameter. Green stands for fulfilled, red for not fulfilled and yellow for controversial

The manufacturer’s name was mentioned in only 64% of the advertisements. As this is typically listed alongside the company’s headquarters, this was included in 63% of the advertisements. The name of the drug was mentioned in 67%, while in 24% of the 181 advertisements, the type of product was not specified, and in 8% the mention was formulated in a questionable way. Warnings were missing in 85% of the ads and in 97% no contraindications were mentioned. This may indicate that these preparations did not require a warning or had no contraindications. Furthermore, only 52% of the advertisements told the reader which indication the medicine was intended for, while 43% presented the application information in a controversial way. As noted earlier, the text “For risks and side effects, read the package leaflet and ask your doctor or pharmacist” (Zu Risiken und Nebenwirkungen lesen Sie die Packungsbeilage und fragen Sie Ihren Arzt oder Apotheker) is a very important mandatory statement when advertising medical products to laypersons according to the HWG. However, this statement was omitted in 39% of the advertisements. Lastly, the composition of the medicinal products was provided in 91% of the advertisements.

Table 3 addresses the prohibitions outlined in the HWG. The prohibitions can be found in Sections 3 and 3a. The prohibitions in a medical advertisement include the following (Fritsche 2021): claims of 100% success rate, no scientific testimonials, physical injury if the drug is not used, pictorial representation of physical injury if the drug is not used, contests, discount promotions or vouchers, and promises of a cure. Just one advertisement mentioned a guaranteed cure if the product was used. Only 33 advertisements referenced studies related to the product or one of its active ingredients. In addition, 99% of the advertisements did not suggest that physical injury could occur if the drug was not used. In line with this, only 4 advertisements contained a clear pictorial representation of bodily injury without applying it to the advertised medicine. Prices, discount promotions and vouchers were found in 2 of the 181 advertisements. An important point of the bans was the promise that the product would cure the disease, as many advertisements contain sentences such as “according to scientists” or “manufactured according to German quality values,” which can lead the patient to assume that the product will be effective. As a result, it was found that 80% of the advertisements contained some kind of promise of a cure.

**Table 3:**
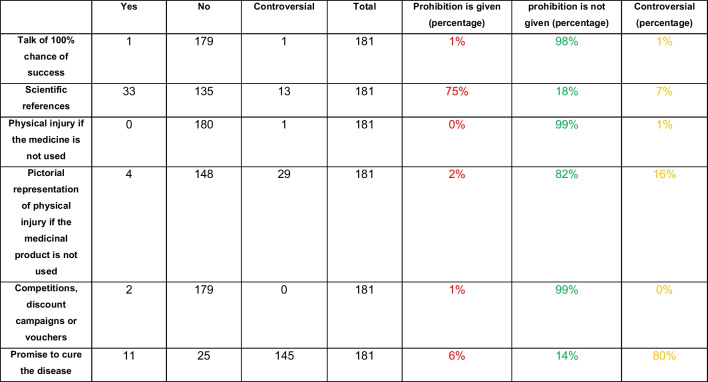
Occurrence of prohibitions according to the HWG. Absolute numbers and percentages are shown for each parameter. Green stands for no prohibition, red for recognizable prohibition and yellow for controversial

## Discussion

### Indication areas in relation to readership

The readership of the HAZ is limited to a certain group of society that is not representative of the entire population. Both the newspaper and the advertisers who choose to promote their products in HAZ are aware of this. Readers are typically over 50 years old, earn over € 3,000 per month and are either employed or retired (ma Tageszeitungen 2022). Accordingly, it makes sense for manufacturers who want to specifically reach this group of readers to advertise their products in the HAZ. Additionally, companies tend to advertise products aimed at treating diseases with a high prevalence in the German population. This can be seen very clearly from the indications of the preparations advertised in the HAZ in 2021 (Fig. 2). Joint pain was the indication with the most advertisements. “57.9% of women and 52.2% of men state that they have had joint pain in the last 12 months” (Fuchs and Prütz 2017). Another frequent indication was back and nerve pain with 33 reports. This is a condition that is very common in the German population, as “61.3% of people in Germany state that they have had back pain at least once in the last twelve months” (von der Lippe et al. 2021). Figure 2 also shows the indications with the fewest advertisements in 2021. Sleep deprivation with a single advertisement generally does not have the highest prevalence compared to other diseases in the German population. “The prevalence of insomnia in adults in Europe is currently between 5.8% and 34.8%, with slightly less than 10% of the population (approx. 6 million people) currently affected in Germany” (Heidbreder 2023). It is therefore conceivable that there are few advertisements for this indication. All in all the manufacturers want to target the HAZ reader group with certain very commonly occurring indications of their products.

### Daily therapy costs

An accurate determination of the daily therapy costs for each individual product was impossible due to the different prices of each provider, which is why https://www.shop-apotheke.com/ was chosen as the source for the prices. As already mentioned, price advertisements are prohibited in an advertisement according to §3 of the HWG (Fritsche 2021).Table 3 indicates that 99% of the ads did not show any price promotions, discounts or vouchers. It was also impossible to calculate the daily cost of creams and gels, as the dose of these dosage forms can vary greatly from one consumer to another. The calculations for the daily treatment costs in this study were roughly estimated but provide a good overview of indications and preparations with the most expensive or cheapest daily costs. The DDD specified by the manufacturer were included in the calculation; these were generally lower for chronic treatment than for acute therapy. As a result, the daily treatment costs for chronic treatment were on average lower than for acute treatment with the same preparation (Figs. 7 and 8).

A product with the indication “sexual weakness” had the highest daily therapy costs for both an acute dose and a chronic dose. The readership of HAZ consists of 52% men and 69% readers who are over 40 years old, who tend to belong to an upper class of society (ma Tageszeitungen 2022).

A 2010 study published in “the Journal of sexual medicine” examined 3,369 men between the ages of 40–79. The subjects were randomly selected from 8 European countries. The study shows that around 30% of the people examined suffer from erectile dysfunction. Men between the ages of 50–59 are the most concerned about the condition (Corona et al. 2010). Accordingly, it makes sense that a drug with this indication could be well received by the HAZ target group.

It is apparent that the calculated daily therapy cots are quite high. Table 4 highlights the products with the top ten highest acute daily therapy costs, comparing them to the costs of their recommended pharmacological alternatives based on the AVR from the year 2023 (Ludwig et al. 2023). It is striking that all the rational pharmacological alternatives exhibit much lower daily therapy costs than the products found in the ads, highlighting that the companies advertising in the HAZ financially exploit perceived medical needs among their readers/customers. The costs for the products advertised in the HAZ are not being made transparent, let alone the comparison with rational alternatives.

**Table 4:** A comparison of the products with the top ten highest daily therapy costs versus the costs of their recommended pharmacological alternatives based on AVR 2023 (Ludwig et al. 2023)

Products	Indication	Daily therpay costs	Rational pharmacological alternative	Daily therapy costs of the alternatives
Product 1	Sexual weakness	9.90 €	Sildenafil (erctile dysfunction)	1.37 €
Product 2	Migraine	5.50 €	Ibuprofen	0.71 €
Product 3	Irritable stomach	4.02 €	Omeparzole (gastroesophageal reflux disease)	0.64 €
Product 4	Nerve/back pain	3.49 €	Pregabalin (neuropathic pain)	1.00 €
Product 5	Water retention and leg pain	3.36 €	Hydrochlorothiazide (HCT)	0.88 €
Product 6	Strained muscles	2.90 €	Methocarbamol (controversial effectiveness)	0.75 €
Product 7	Sexual weakness	2.47 €	Sildenafil (erectile dysfunction)	1.37 €
Product 8	Irritable bowel and diarrhea	2.40 €	Mebeverin (spasmolytic effect)	1.01 €
Product 9	Dizziness	2.37 €	Betahistine (controversial effectiveness)	0.46 €
Product 10	Joint pain	2.25 €	Diclofenac (short-term use in osteoarthritis and injuries)	0.50 €

### Scientific evidence of the advertisements

As mentioned previously, the HWG prohibits advertising medical products whose effects lack a scientific basis (Fritsche 2021). It emerged that studies were mentioned in only 46 of the advertisements (Fig. 9a), and in 13 ads, these studies were unrelated to the advertised drug (Fig. 9b). In addition, several advertisements contained texts that are very difficult for the average reader to evaluate, such as “according to scientists,” “studies have shown,” and “manufactured according to German quality standards.” The 33 advertisements that referenced studies specifically related to the product or one of its active ingredients also required careful evaluation. However, studies generally referred to an active ingredient rather than to the drug itself.

Figure 10 shows that 15% of the available studies analyzed fell into evidence classes 4, 3, and 2b. Accordingly, the remaining 85% of the 33 studies were classified within the three higher evidence classes. However, it is important to note that the same studies are often reused in multiple ads for the same drug to enhance its credibility across different ads. For this reason, the individual 39 products of the 181 advertisements and their active ingredients were checked for PubMed articles. Figure 9c shows that an article was found for only 60% of the products. Only one article referred to the drug itself. 

In summary, most advertisements did not mention studies, and for those that did, it was very difficult to evaluate the studies accurately. For a layperson, it would be nearly impossible to discern whether the studies cited in the ads are reliable. In addition, certain phrases such as “dermatologically tested” or “according to studies” can attract the reader’s attention without necessarily having a robust scientific basis behind them.

### Therapeutic Products Advertising Act

We developed a traffic light system to assess advertisements for compliance with the HWG. This was necessary because both the HWG and the advertisements contain very loosely formulated phrases, making it impossible to determine whether the advertisements comply with the law. This is indicated by the yellow color in the two tables. This was also the case in the Apotheken Umschau study (Keuper and Seifert 2024). Table 5 illustrates as a summary the overall implementation of the HWG in comparison to the Apotheken Umschau study, considering both the required mandatory information and the prohibitions under the HWG for the calculation.

**Table 5:**
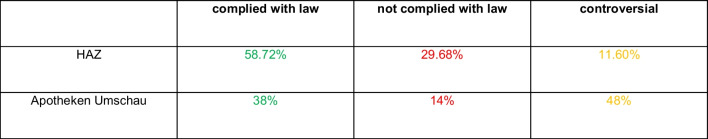
Comparison of the implementation of the HWG between HAZ and Apotheken Umschau, Green stands for no prohibition, red for recognizable prohibition and yellow for controversial. A summary of all criteria analyzed is presented. The individual percentage values were added together and divided by the total sum of percentages, resulting in the average

Using Table 2, it can be calculated as a percentage that mandatory information was included in 52% of the advertisements, omitted in 41%, and presented ambiguously in 7%. In almost every advertisement the mandatory information appeared in very small text below the advertisement. In 66 advertisements, the name of the manufacturer and in 67 advertisements the registered office of the company were not mentioned likely to avoid direct consumer contact.

The limited mention of warnings and contraindications was surprising. Only one ad mentioned a contraindication, and 22 ads had warnings, potentially suggesting to readers that most products can be used without risks. In addition, phrases like “No known interactions or side effects” or “97% of patients had no interactions or side effects” appeared, without specifying the potential interactions or side effect, which may mislead consumers. The fact that the prices of the advertisements in the HAZ depend on the size of the advertisement (https://www.haz.de/media/price list) means that the manufacturers only have limited space to persuade readers about their products. This often leads to the omission of critical information, especially important for medical products.

Table 3 addresses HWG prohibitions. 69% of the advertisements avoided prohibited elements, 14% contained prohibitions and 17% were controversial. Overall, conspicuous claims that might imply the product is a “miracle cure” were generally avoided, as none of the ads indicated physical injury if the medicine was not used, and 6% of the ads promised to cure the disease (Table 3). There were no scientific studies in 135 advertisements, as Fig. 9a shows. The scientific basis is the cornerstone of medical products, and the layperson has the right to be informed whether the advertised products and its effects have been proven by studies. Price promotions, discount campaigns or vouchers, which the HWG prohibits, were nevertheless present in only 2 advertisements.

In general, the 181 ads avoided more HWG prohibitions than they provided mandatory information. The HWG is very loosely formulated at several points and is subject to very little control. This lack of enforcement was already highlighted by a study conducted by Arzneimittelbrief in 1999 (Meyer 1999), in which 861 advertisements were examined with regard to all relevant elements of the HWG. The study showed that the provisions of the HWG were not implemented in more than 80% of the advertisements. The implementation of the law is apparently not only to be criticized in print newspapers but also in other media, as the study on advertising in online pharmacies shows (Barlage and Seifert 2024).

## Limitations of the study

This study was conducted with public sources. The evaluation of the studies mentioned in the advertisements was very difficult and partly subjective. Due to the wording of the law and the wording of the texts in the advertisements, the implementation of the HWG in certain cases was based on the individual opinion of the authors, which is why the yellow column in Tables 2 and 3 was very important. Only advertisements from 2021 were analyzed, making a comparison with other years impossible. It should also be mentioned that advertisements for the same preparation appeared several times during the year. For the most part, the ads had the same content and the same text. Nevertheless, these had to be analyzed as individual advertisements, as sometimes the content differed minimally. The calculation of the daily therapy costs was only possible to a limited extent.

## Conclusions and outlook

The present study shows that the medical advertisements in the HAZ do not prioritize the health of laypersons. Rather, marketing and strategic factors dominate the advertisements, like it was the case even in professional journals (Kuschel and Seifert 2024). A dominant factor in this study is the price that manufacturers must pay to advertise their products in the HAZ, which is why the ads were only found during the week. The HAZ target group is obviously considered by the companies. This can be seen very clearly in the indications and areas of application of the drugs. The study also shows that the handling of evidence-based medicine and science was very questionable in many advertisements. This can be seen from the fact that most of the preparations were homeopathic. The few advertisements in which studies were mentioned did not deal with the actual medicine, but rather with an active ingredient. The fact that there were hardly any warnings, contraindications, interactions or side effects in the text of the advertisements is very striking. This all indicates that the monitoring of the implementation of the HWG is not functioning properly. The loose wording of the HWG, which is confusing at certain points, allows the existence of a gray area that can be exploited for financial purposes. Better control of the HWG and separate regulation of medical advertisements in newspapers could make the task easier for both the reviewing authorities and the magazines themselves. Further studies therefore need to be carried out, looking at other years and other newspapers. The small text containing the name of the company, company headquarters and active ingredients should be written larger. From a pharmacological point of view, there is room for improvement as well. Doctors and pharmacists should check their patients’ medical histories very carefully and bear in mind that advertising in the various advertising media is increasingly being used for medical products that are only intended for financial reasons. Newspapers should also be aware of this, which is why they should develop better checking mechanisms for medical advertisements.

## Take-home messages

- To our knowledge, this is the first study medical advertisements in a German daily newspaper.
- Medical advertisements are influenced by the newspaper’s reader group.
- Scientific studies on the effects of a product are rare.
- The Therapeutic Products Advertising Act is not properly implemented in many advertisements.
- Lay people, doctors and pharmacists need to be informed about the extent to which commercial and psychological aspects play a role in medical advertisements.

## Data Availability

All source data for this work are available upon reasonable request.

## Abbreviations

HAZ: Hannoversche Allgemeine Zeitung (The Hannover General Newspaper)
HWG: Heilmittelwerbegesetz (Medicines Advertising law)
DTCA: Direct-to-consumer-advertising
DDD: Defined daily doses
AVR: Arzneiverordnungsreport (drug prescription report)
RCT: Randomized controlled trials
